# Duodenal stenosis, an unusual presentation of eosinophilic gastroenteritis: a case report

**DOI:** 10.3389/fped.2024.1390946

**Published:** 2024-04-18

**Authors:** Clelia Di Mari, Elena Pozzi, Cecilia Mantegazza, Francesca Destro, Milena Meroni, Marina Coletta, Andrea Sorge, Gloria Pelizzo, Gian Vincenzo Zuccotti

**Affiliations:** ^1^ Department of Pediatrics, Buzzi Children’s Hospital, Milan, Italy; ^2^ Department of Pediatric Surgery, Buzzi Children’s Hospital, Milan, Italy; ^3^ Gastroenterology Unit, Fondazione IRCCS Ca’ Granda Ospedale Maggiore Policlinico, Milan, Italy; ^4^ Department of Pathophysiology and Transplantation, University of Milan, Milan, Italy; ^5^ Department of Biomedical and Clinical Sciences, University of Milan, Milan, Italy

**Keywords:** eosinophilic gastroenteritis, stenosis, surgical treatment, quality of life, case report

## Abstract

Eosinophilic gastrointestinal diseases (EGIDs) are rare, chronic inflammatory disorders characterized by eosinophilic infiltration of the gastrointestinal tract. Symptoms and clinical presentations vary depending on the site and layer of the gastrointestinal wall infiltrated by eosinophils. Gastrointestinal obstruction is a serious, though uncommon, presentation. Management can be extremely challenging because of the rarity of the condition and the lack of robust scientific evidence. Current treatment approaches for EGIDs mainly focus on elimination diets, proton pump inhibitors and corticosteroids, which present high refractoriness rates. Novel targeted therapies are being investigated but not routinely used. Surgery should be avoided as far as possible; however, it may be the only option in gastrointestinal obstruction when long-term remission cannot be attained by any medical strategy. Herein we report the case of an adolescent boy affected by an eosinophilic gastrointestinal disease with progressive duodenal stenosis, refractory to medical therapy, who successfully benefitted from surgical management. He presented with a one-year history of gastrointestinal obstructive symptoms with feeding intolerance. After the diagnostic workup, he was diagnosed with an eosinophilic gastrointestinal disease (esophagitis and enteritis) with a duodenal involvement causing a progressive duodenal stenosis. Due to refractoriness to the conventional medical therapies and the consequent high impact on his quality of life, related both to the need for enteral nutrition and repeated hospitalizations, we decided to perform a gastro-jejunum anastomosis, which allowed us to obtain a clinical and endoscopic long-term remission. The early discussion of the case and the involvement of all experienced specialists, pediatricians and pediatric surgeons is essential.

## Introduction

Eosinophilic gastrointestinal diseases (EGIDs) are a group of rare, chronic inflammatory disorders characterized by primary eosinophilic inflammation of specific segments in the gastrointestinal (GI) tract (1, 2). Multiple GI segments may be simultaneously or sequentially involved (1).

EGIDs' diagnosis is based on symptoms associated with histological findings of intestinal eosinophilic infiltration after excluding a secondary cause of tissue eosinophilia. Symptoms differ according to the patient's age and the localization, extension, and depth of the eosinophilic infiltration through the intestinal wall. Mucosal disease is the most common form and presents with non-specific symptoms such as nausea, vomiting, diarrhea, failure to thrive, dysphagia, dyspepsia, abdominal pain, and gastrointestinal bleeding. The serosal form occurs in a minority of patients and is characterized by exudative eosinophil-rich ascites, bloating and abdominal pain (1). Patients with muscular involvement, often affecting the stomach and duodenum, may develop intestinal obstruction or sub-obstruction because of the eosinophilic inflammation and fibrosis of the muscular layer of the bowel (3).

Histological confirmation of EGIDs diagnosis may be challenging since uniformly accepted histological criteria for EGIDs, beyond Eosinophilic Esophagitis (EoE, ≥15 eosinophils per high power field, HPF), are still debated (1, 4). Collins and colleagues in 2018 proposed the following criteria for the histological diagnosis of non-EoE EGIDs: diagnosis of Eosinophilic Gastritis (EoG) includes an eosinophilic count ≥30/HPF in more than 5 HPF and ≥70/HPF in more than 3 HPF; diagnosis of Eosinophilic Enteritis (EoN) and Eosinophilic Colitis (EoC) includes an eosinophilic count at least double the peak number found in normal biopsies (EoN: >52/HPF in duodenal mucosa and >56/HPF in ileum; EoC: >100/HPF in cecum and right colon, >84/HPF in transverse and left colon, >64/HPF in rectum and sigma) (5).

Eosinophilic Esophagitis (EoE) represents the most known and common form, with a prevalence of 1/2,000 subjects (6). Eosinophilic Gastritis (EoG), EoN and EoC are rarer forms (2, 7) whose epidemiology and pathophysiology are still unclear (4). According to different cross-sectional studies, the prevalence of the latter three forms ranges, respectively, from 1.5 to 6.4/100.000, from 2.7 to 8.3/100.000 and from 1.7 to 3.5/100.000 subjects (8). Nevertheless, accurate data on incidence and prevalence of non-EoE EGIDs are difficult to establish because most of them derive from case reports and small retrospective series; moreover, in many case series, the diagnosis of EGID was based on non-standardized eosinophils cut-off values. Recently, an Italian multicenter study retrospectively described a cohort of 40 adult patients affected by EoC, which currently represents the largest reported cohort of EoC by adopting the stringent histological cut-off values proposed by Collins et al. (9).

First-line treatment approaches focus on elimination diets, proton pump inhibitors (for upper EGIDs), and topical and systemic corticosteroids. However, due to standard therapy refractoriness and high recurrence rate, effective maintenance treatments are needed both in adult and pediatric patients (10–13). Immunomodulators (e.g., azathioprine), sodium cromoglycate, or montelukast have been tried as maintenance therapy, but with poor efficacy (14–16). Novel targeted therapies, including monoclonal antibodies (targeting IL-13, IL-4, IL-5, integrins, Siglec-8) and non-biological treatments (targeting JAK-STAT and CHTR2 signaling pathways) seem to be promising and are currently being investigated, although not routinely used (14, 16–18).

Little has been published about the surgical management of EGIDs. Surgery may sometimes be required when complications like perforation or obstruction occur (19–21). It has been reported that about 40% of EGID patients may need surgery and about half of them may experience recurrence after surgery (22).

Gastrointestinal obstruction represents an unusual presentation of EGID and is generally associated with predominantly muscular disease. In infants, gastric outlet and duodenal strictures may mimic pyloric stenosis (3, 23, 24). More distal intestinal obstructions, although rare, have been described and may occur in the ileum (3) and jejunum (25). Obstructive symptoms are mostly reversible under corticosteroids. Surgery is reserved for recurrent forms when long-term remission cannot be obtained by any medical strategy (26, 27).

Diagnosis and treatment of EGIDs remain very challenging in the case of muscular and serosal involvement. Moreover, the chronic nature of the disease, long-term therapies, and strict follow-up may impair the quality of life of patients and their families (28–30).

Herein, we describe the case of an adolescent boy presenting with esophageal and small bowel EGID, complicated by a progressive duodenal stenosis, who failed to respond to long-term medical treatment and benefitted from surgical management. We want to highlight the diagnostic and therapeutic challenges, the importance of a strict, early, and continuous collaboration between pediatricians and pediatric surgeons, and the delicate and complex aspects of follow-up.

## Case presentation

A 14-year-old boy was referred to our Pediatric Gastroenterology Unit at Vittore Buzzi Children's Hospital in Milan in June 2018, with a one-year history of upper abdominal pain, repeated nonbilious vomiting, and occasional dysphagia. Five kilograms were lost yearly; no diarrhea or gastrointestinal bleeding were reported. He had suffered from recurrent aphtous stomatitis and allergic oculorhinitis from age 8. Neither allergic asthma nor chronic rhino-sinusitis with polyposis were associated. His family history included allergic asthma and psoriasis.

At referral, his weight was 56 kg (0 SD); his height was 165 cm (0 SD). His clinical examination was unremarkable, except for upper abdominal tenderness.

Blood tests revealed mild eosinophilia (Eo 1,000/mm^3^) and increased total IgE (341 kU/L). Erythrocyte sedimentation rate (ESR), C reactive protein (CRP), liver and pancreatic tests were normal; serum albumin was normal. Fecal calprotectin and parasitological examination resulted negative. Skin prick tests were positive for some inhalants, and negative for food allergens.

Abdominal ultrasound showed dilation of common hepatic, common bile, cystic and pancreatic ducts. The magnetic resonance cholangiopancreatography confirmed the dilation of the biliary tract, while the pancreas resulted normal. A congenital malformation of the biliary tree (choledochal cyst) was hypothesized. Moreover, a thickening of the wall of the descending duodenum was observed and confirmed by a magnetic resonance enterography. No other involvement of small bowel was detected.

**Figure 1:**
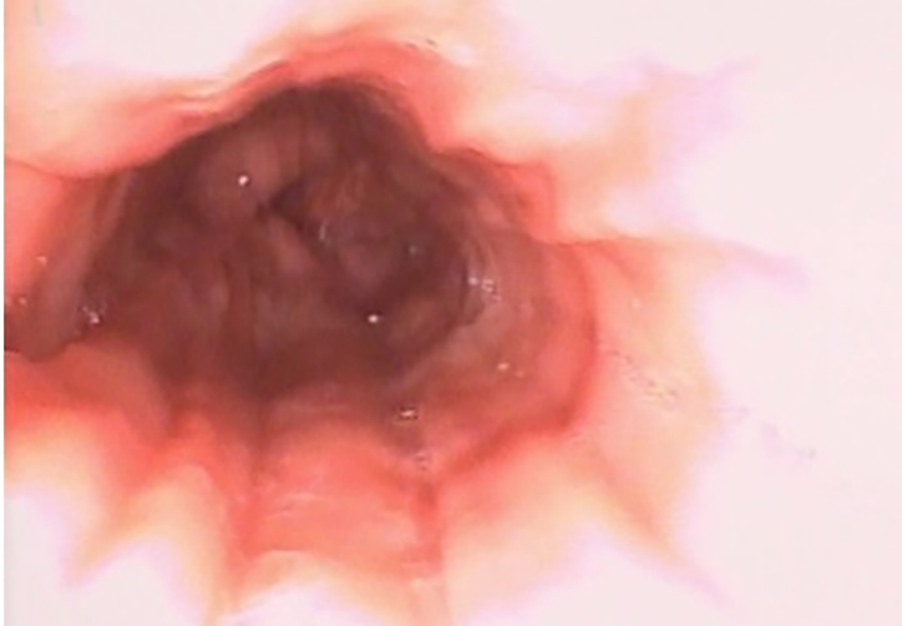
EGD documenting esophagitis in the 3rd esophageal tract characterized by reduced wall distensibility, trachealization and furrows.

**Figure 2:**
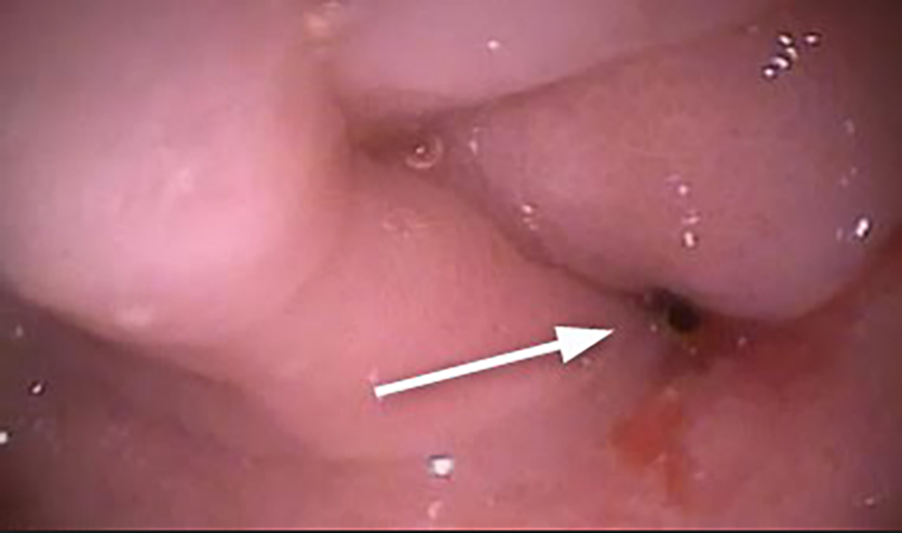
Duodenal stenosis (arrow): edema in the duodenum prevented the progression of the 9 mm endoscope. A 5 mm endoscope was used to overcome the stenotic tract.

**Figure 3:**
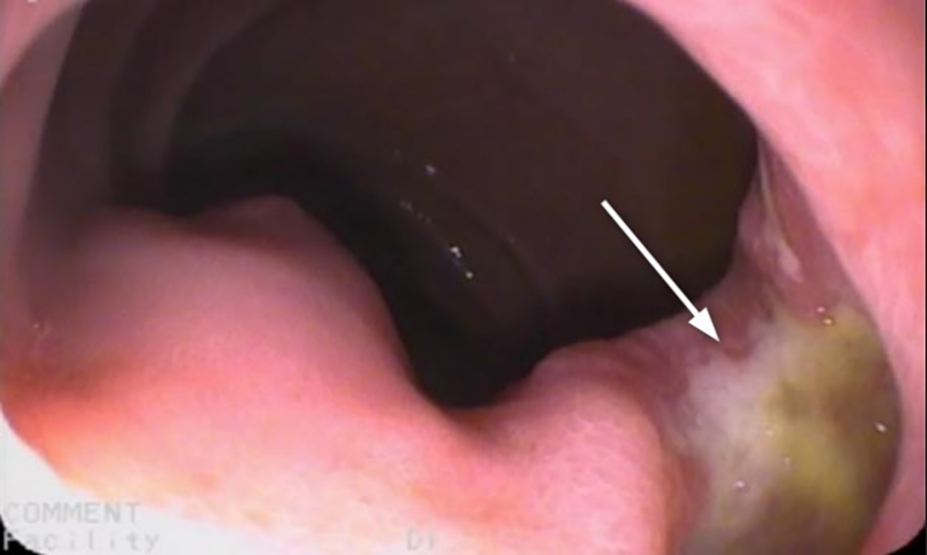
Post-stenotic juxta papillary ulceration (arrow) was identified with no active bleedings.

Esophagus-gastro-duodenoscopy (EGD) revealed a pale, mild trachealized esophagus with longitudinal furrows (Figure 1), normal gastric mucosa, and the presence of an edematous duodenal bulb associated with a tight stenosis of the descending duodenum (Figure 2); a neonatal endoscope was effective in overcoming the narrowing, and a post-stenotic juxta papillary ulceration was identified (Figure 3). Ileocolonoscopy was normal.

Eosinophilic inflammation was found in all the biopsies, but the gastric ones: >60 eosinophils/high power field (HPF) in the terminal ileum and colon, >30/HPF in the duodenum, >15/HPF in all the esophageal tracts; clusters of eosinophils were detected with focal epithelial infiltration. Neither parasites nor tumoral cells, morphological elements attributable to inflammatory bowel diseases or other microscopic colitis were identified.

The final diagnosis was EGID: eosinophilic esophagitis and enteritis; we could not confirm a colonic involvement due to the lack of assessment of the precise eosinophilic count in biopsies, as compared to the criteria proposed by Collins and colleagues (5), and the absence of symptoms suggestive of colitis.

A course of systemic steroids (prednisone 40 mg/day for 2 weeks, then tapered in 10 weeks) was prescribed, associated with proton pump inhibitors (PPI) (esomeprazole 40 mg/day), with a prompt clinical response. The follow-up EGD, performed after 4 weeks of treatment, showed a global improvement: the esophagus was easily distensible without trachealization and only mild exudate; the descending duodenum stenosis, though still present, was passable by a 9 mm endoscope; the duodenal mucosa in the post-stenotic tract appeared repaired. Histological examination documented the absence of eosinophilic inflammation in all the examinated fragments.

As a maintenance treatment, a 3-food (milk, egg, and wheat) elimination diet was then prescribed; however diet therapy success was limited by poor patient adherence.

**Figure 4:**
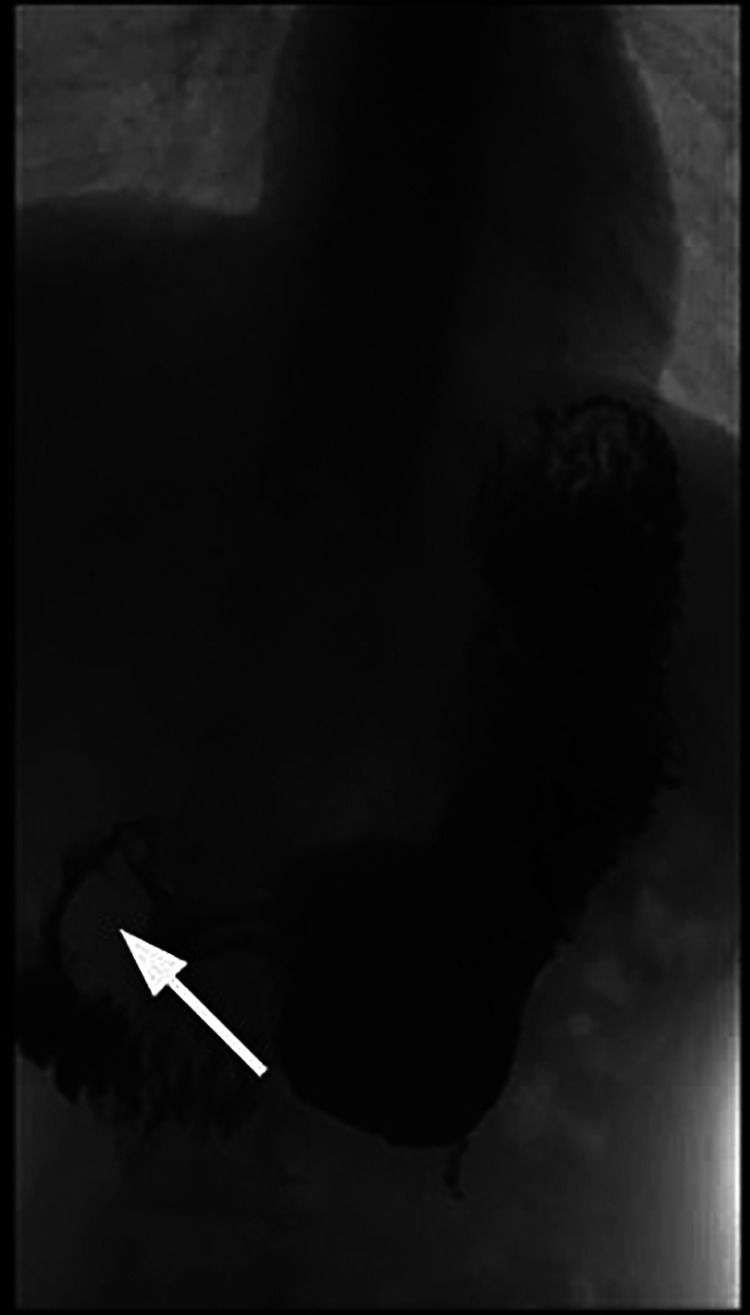
Duodenal stenosis (arrow) confirmed by upper GI series.

The patient experienced a clinical relapse 1 month after the discontinuation of steroids; the upper GI series confirmed the recurrence of duodenal stenosis (Figure 4).

Therapy with systemic steroids and PPI was successfully restarted and associated with azathioprine (2 mg/kg/day) as a maintenance treatment. Oral budesonide was started at prednisone tapering.

A 3-month-follow-up EGD revealed a passable duodenal stenosis. Histological examination of gastric and duodenal biopsies revealed the absence of eosinophilic inflammation.

A third relapse occurred six months later, requiring a course of intravenous methylprednisolone; a maintenance therapy of montelukast 10 mg/day was associated with azathioprine.

Due to oral feeding intolerance and weight loss, a total pre-pyloric enteral nutrition with hydrolyzed formula was started and administered by nasogastric tube.

The magnetic resonance enterography showed a worsening in duodenal wall thickening; moreover, gastric wall thickening was encountered, suggesting an eosinophilic muscular infiltration of the stomach. A non-passable stenosis was confirmed by EGD.

Given the ineffectiveness of medical therapy in preventing the recurrence of duodenal stenosis, with consequent persistent oral feeding intolerance and the high psychological impact related to enteral nutrition and repeated hospitalizations, we decided to perform, in September 2020, a video-laparoscopic side-to-side gastro-jejunum anastomosis by a stapler device without any resection, to obtain a surgical bypass of the obstructed duodenum. Endoscopic dilatation of the duodenal stenosis was not technically possible because of the proximity to the biliary papilla. Unfortunately, it was impossible to take full-thickness biopsies of the thickened duodenal bulb and stomach because of the extreme stiffness of the wall.

Surgery effectively relieved the patient's symptoms, allowing a gradual withdrawal of enteral nutrition. Medical therapy with montelukast and oral budesonide was continued while azathioprine was suspended because of hematological side effects. Follow-up endoscopies, performed 6 and 18 months after the surgical treatment, showed an improvement in the duodenal stenosis, highlighting a possible beneficial effect of partial bowel diversion on EGID remission. After the 18-months-follow-up EGD, budesonide was suspended while therapy with montelukast is still ongoing, associated with omeprazole. Follow-up magnetic resonance enterography, performed 36 months later, showed stable duodenal involvement without pre-stenotic dilatation. Follow-up EGD is scheduled.

No clinical relapses occurred 36 months after surgery with a great amelioration and normalization in the patient's quality of life.

## Discussion

EGIDs’ long-term outcome has yet to be fully characterized (31). In a retrospective study conducted on 43 EGIDs patients over a follow-up period of 13 years, 42% of patients experimented no clinical relapse after the initial flare. In 37% of patients, multiple flares alternated with periods of full remission while 21% presented a chronic disease course (32). Another study, conducted on 44 pediatric and adult EGIDs patients, reported that only one-third remained in remission after a mean follow-up of 26.2 months, while most patients presented a persistent or progressive disease course. Better response rates were showed for oral corticosteroids than for elimination diets, leukotriene antagonists, H2 blockers and mast-cell inhibitors (33).

Oral systemic steroids have been shown to induce both clinical remission and a reduction in mucosal eosinophilia (34). Topical steroids (fluticasone, budesonide), commonly used for EoE, may be considered in EGIDs. Their efficacy in reducing symptoms and tissue eosinophilia has been reported in some retrospective studies (35–37).

However, relapses may occur at discontinuation of steroids. The chronic relapsing course of EGIDs, the need for restrictive diets, multiple medical treatment changes, and the frequent endoscopies needed during follow-up may hugely impact the quality of life of EGIDs patients (29, 30, 38), indicating the need for long-term maintenance therapies.

Sodium cromoglycate, montelukast and immunomodulators such us azathioprine demonstrated poor efficacy as maintenance therapies (14–16).

Novel biological drugs, targeting T helper 2 (TH2) (IL-25, IL-33, TSLP, IL-4, IL-5, IL-9, IL-13) and non-TH2 pathways, are being investigated to treat EGIDs. Dupilumab, an anti-IL-4 receptor alpha (IL-4Rα) monoclonal antibody, blocking IL-4 and IL-13 signaling, is already approved for atopic dermatitis in children older than 6 months, for asthma in children older than 6 years, and for nasal polyposis in adults. In May 2022, Dupilumab has been approved for EoE in patients older than 12 years (39). Efficacy of Dupilumab is currently being investigated in a phase 2 multi-center trial in adults and children older than 12 years affected by EoG (ClinicalTrials.gov Identifier: NCT03678545). Anti-IL-5 monoclonal antibodies (Reslizumab, Mepolizumab) have been demonstrated to significantly reduce the esophageal eosinophilic inflammation in children affected by EoE, but no difference emerged in reducing symptoms. Their efficacy in treating non-EoE EGIDs in adults and in adolescents is currently under investigation (40, 41). Lirentelimab is an antibody targeting the sialic acid-binding immunoglobulin-like lectin 8 (Siglec-8), which induces the apoptosis of activated eosinophils; in a phase 2 clinical trial conducted on adult patients affected by EoG and eosinophilic duodenitis, it was demonstrated to reduce gastrointestinal eosinophilia and symptoms (42). Vedolizumab, an α4β7 integrin inhibitor used in the treatment of inflammatory bowel diseases, may improve eosinophilic infiltration and reduce steroid dependency in refractory EoG and EoN (43).

A recent retrospective multicenter study, conducted both in children and adults affected by EGIDs, reported that only 2/142 (1%) patients with EoG, 4/123 (3%) patients with EoN, and 1/108 (1%) patients with EoC, were treated with a monoclonal antibody, with clinical, endoscopic, and histologic improvements in short term follow-up (34).

The European and North American Societies for Paediatric Gastroenterology Hepatology and Nutrition (ESPGHAN and NASPGHAN), in their recent joint guidelines, recommend considering oral systemic steroids to induce remission in children with non-EoE EGIDs; they confirm the lack of sufficient evidence to recommend for or against the use of leukotriene inhibitors, mast cell blockers, immunomodulators and biologics in this group of patients; they consider the possibility to evaluate the use of proton pump inhibitors in children affected by EoG or eosinophilic duodenitis with ulcerations and the use of topical steroids and empiric elimination diets in selected patients (44).

Little has been published about the surgical management of EGIDs. Sheick and colleagues described five cases of EGID-related gastrointestinal obstruction, with predominant involvement of the stomach and duodenum. Four out of 5 patients benefitted from medical treatment. A 71-year-old woman in their series was affected by eosinophilic gastritis, with thickened mucosal folds and multiple antral polypoid lesions causing recurrent gastric outlet obstruction. She failed to respond to steroids and sodium cromoglicate and successfully underwent antrectomy and gastrojejunostomy with consequent remission on low-dose corticosteroids and sodium cromoglicate (26). Shetty and Shetty reported two cases of recurrent subacute intestinal obstruction whose diagnosis of EGID was made after surgery; they both recovered well (27).

Given the rarity of complicated EGIDs, treatment strategies are adjusted each time, relying on empiric considerations and personal experience rather than evidence. There is no consensus or guidelines for treating patients with complex EGIDs forms, considering that the disease's pathogenesis is largely unclear.

The main concerns about surgery of EGIDs are the intervention timing, the execution of minimally invasive and reversible procedures, and the adequacy of the follow-up. Treatment decisions should be made on the extension of the gastrointestinal involvement and clinical severity.

Laparoscopy plays an important role in the diagnosis of EGID when a muscular or serosal involvement is suspected, since it allows us to perform full-thickness biopsies to prove the eosinophilic infiltration, which cannot be detected by endoscopic mucosal biopsies (45). The approach is minimally invasive and well tolerated even in pediatric ages.

Surgery may be required in complicated and life-threatening conditions, such as intestinal perforation or intussusceptions (19–21). It could also be needed in recurrent EGID-related obstructions, which are not reversible under corticosteroids; furthermore, in some uncertain cases, the histopathology of the surgically removed segments can confirm the diagnosis definitively (3, 45, 46).

Our patient presented with a duodenal stenosis, an unusual manifestation of the disease. The histopathologic evaluation of biopsies, negative for morphological elements attributable to inflammatory bowel diseases, other microscopic colitis or for tumoral cells, allowed us to rule out Crohn's disease and neoplasms. Patient medical history, parasitic tests on both stools and biopsies and an allergologic evaluation allowed for the exclusion of a secondary cause of tissue eosinophilia.

Our patient benefitted from a combined medical and surgical management of his condition, with a good outcome, similarly to other cases already reported in the literature (26, 27). As limitations, we could not accede to novel targeted treatments because of a lack of authorization in our pediatric center at the time of patient evaluation. Our case report highlights the role of surgery in progressive intestinal stenosis in EGID. Given the high risk of recurrence, it raises the need for a proper follow-up, notably with surveillance of the bind-ending intestinal loop and maintenance treatment.

## Conclusion

EGIDs are rare, chronic relapsing conditions for which a high degree of clinical suspicion is necessary for diagnosis. Therapeutic approaches are still inadequate and poorly standardized, especially in maintaining remission (1). Surgery should be avoided as far as possible (4). Still, it can be useful for managing recurrent intestinal obstructions if medical treatments cannot attain long-term remission. A “more intense” treatment, including immunomodulators, targeted therapies, and endoscopic and/or surgical procedures, may be required in complicated disease. Each case should always be discussed between all the pediatric experts, starting from the initial stages of care, to ensure the best-tailored management and establish the proper intervention timing.

## Data Availability

The original contributions presented in the study are included in the article, further inquiries can be directed to the corresponding author.
